# Supplementary material for: Cardiovascular Mortality During the COVID-19 Pandemics in a Large Brazilian City: A Comprehensive Analysis
Source: Glob Heart. 2022 Feb 21;17(1):11. doi: 10.5334/gh.1101 (PMC8877643; doi:10.5334/gh.1101)
Supplement: Supplementary Material 1. — Percent of hospital admissions processed in each month from the total of admissions in the respective month in 2019 and 2020, Hospital Information System, Belo Horizonte, MG, Brazil. [file gh-17-1-1101-s1.pdf]

Supplementary Material 1: Percent of hospital admissions processed in each month from the total of admissions in the respective month in 2019 and 2020, Hospital Information System, Belo Horizonte, MG, Brazil.

		Processing Month																
		2019-01	2019-02	2019-03	2019-04	2019-05	2019-06	2019-07	2019-08	2019-09	2019-10	2019-11	2019-12	2020-01	2020-02	2020-03	2020-04	2020-05
Hospital Admission Month	2019-01	32.32	41.01	16.58	8.25	1.24	0.36	0.06	0.02	0.02	0.02	0.02	0.01	0.01	0.01	0.01	0.01	0.01
	2019-02	0	33.1	43.07	14.85	7.32	1.33	0.29	0.03	0	0.01	0	0	0	0	0	0	0
	2019-03	0	0	33.81	40.45	16.94	7.15	1.23	0.32	0.07	0.02	0.02	0	0	0	0	0	0
	2019-04	0	0	0	37.23	37.78	16.01	7.54	1.01	0.32	0.05	0.01	0.01	0.01	0.01	0	0.01	0.01
	2019-05	0	0	0	0	35.97	38.93	16.02	7.43	1.19	0.3	0.04	0.01	0.01	0.01	0.01	0.01	0.01
	2019-06	0	0	0	0	0	32.36	45.87	13.17	6.98	1.25	0.34	0.03	0	0	0	0	0
	2019-07	0	0	0	0	0	0	37.18	42.31	12.08	6.87	1.07	0.39	0.09	0.02	0.01	0	0
	2019-08	0	0	0	0	0	0	0	37.62	41.18	12.54	6.46	1.58	0.44	0.07	0.03	0.02	0.02
	2019-09	0	0	0	0	0	0	0	0	35.65	44.73	9.96	7.8	1.39	0.39	0.06	0.01	0
	2019-10	0	0	0	0	0	0	0	0	0	39.51	36.19	12.6	9.99	1.13	0.49	0.06	0.01
	2019-11	0	0	0	0	0	0	0	0	0	0	37.76	34.52	17.56	8.39	1.33	0.37	0.06
	2019-12	0	0	0	0	0	0	0	0	0	0	0	36.4	34.17	20.21	7.75	1.09	0.28
