# Supplementary material for: Cardiovascular Mortality During the COVID-19 Pandemics in a Large Brazilian City: A Comprehensive Analysis
Source: Glob Heart. 2022 Feb 21;17(1):11. doi: 10.5334/gh.1101 (PMC8877643; doi:10.5334/gh.1101)
Supplement: Supplementary Material 3. — Age-standardized rates per 100,000 inhabitants and proportions for ICD Chapter XVIII outcomes observed in 2020, and expected (mean of 2015–2019), for epidemiological weeks 10–48, their absolute difference, and risk ratio. Belo Horizonte MG, Brazil. [file gh-17-1-1101-s3.pdf]

Supplementary Material 3: Age-standardized rates per 100,000 inhabitants and proportions for ICD Chapter XVIII outcomes observed in 2020, and expected (mean of 2015-2019), for epidemiological weeks 10-48, their absolute difference, and risk ratio. Belo Horizonte MG, Brazil.

	Observed	Expected	Absolute Difference	Risk Ratio
Deaths	33 (30;37)	39 (36;43)	-6.4	0.84 (0.73;0.96)*
Deaths at Home	21 (19;24)	25 (22;28)	-3.7	0.85 (0.72;1.01)
% Deaths at Home	64 (59;69)	63 (59;68)	1.0	1.02 (0.86;1.2)
Hosp. Admissions	100 (95;106)	98 92;104)	2.4	1.02 (0.94;1.11)
ICU	20 (18;23)	19 (16;21)	1.7	1.09 (0.9;1.31)
% ICU	20 (18;22)	19 (17;21)	1.2	1.06 (0.88;1.28)
In-Hospital Deaths	7 (5;9)	8 (6;9)	-0.7	0.91 (0.67;1.23)
% In-Hospital Deaths	7 (5;8)	8 (6;9)	-0.9	0.89 (0.66;1.2)

ICD: International Classification of Diseases, ICU: intensive care unit
