# Supplementary material for: Cardiovascular Mortality During the COVID-19 Pandemics in a Large Brazilian City: A Comprehensive Analysis
Source: Glob Heart. 2022 Feb 21;17(1):11. doi: 10.5334/gh.1101 (PMC8877643; doi:10.5334/gh.1101)
Supplement: Supplementary Material 4. — Age-standardized rates per 100,00 inhabitants and proportions for cardiovascular disease outcomes observed in 2020, and expected (mean of 2015-2019) for epidemiological weeks 10–48, their absolute difference, and risk ratio, according to age range. Belo Horizonte, MG, Brazil. [file gh-17-1-1101-s4.pdf]

Supplementary Material 4: Age-standardized rates per 100,00 inhabitants and proportions for cardiovascular disease outcomes observed in 2020, and expected (mean of 2015-2019) for epidemiological weeks 10-48, their absolute difference, and risk ratio, according to age range.Belo Horizonte, MG, Brazil.

Variable	30-59				60+			
	Observed	Expected	Absolute Difference	Risk Ratio	Observed	Expected	Absolute Difference	Risk Ratio
Deaths	50 (46;55)	55 (50;61)	-5.11	0.91 (0.79;1.04)	734 (706;764)	715 (686;744)	19.63	1.03 (0.97;1.09)
Deaths at Home	15 (13;18)	14 (12;17)	1.31	1.09 (0.85;1.4)	253 (236;271)	185 (170;200)	68.20	1.37 (1.23;1.52)*
% Deaths at Home	31 (26;35)	25 (21;29)	5.19	1.2 (0.93;1.54)	34 (32;36)	25 (24;27)	8.59	1.33 (1.2;1.48)*
Hosp. Admissions	387 (374;401)	583 (567;600)	-196.07	0.66 (0.63;0.69)*	1760 (1716;1807)	2123 (2074;2174)	-362.80	0.83 (0.8;0.86)*
ICU	150 (141;158)	175 (166;184)	-25.15	0.86 (0.79;0.92)*	669 (642;698)	751 (722;782)	-82.43	0.89 (0.84;0.94)*
% ICU	38 (37;40)	30 (28;31)	8.69	1.29 (1.19;1.39)*	38 (36;39)	35 (34;36)	2.61	1.07 (1.01;1.14)*
In-Hospital Deaths	14 (12;17)	18 (15;21)	-3.77	0.8 (0.63;1.01)	135 (123;149)	148 (135;162)	-12.69	0.91 (0.8;1.04)
% In-Hospital Deaths	3 (3;4)	3 (2;3)	0.64	1.2 (0.95;1.53)	7 (7;8)	7 (6;7)	0.73	1.1 (0.97;1.25)

ICU: intensive care unit
